# Supplementary material for: Permutation-based significance analysis reduces the type 1 error rate in bisulphite sequencing data analysis of human umbilical cord blood samples
Source: Epigenetics. 2022 Mar 4;17(12):1608–27. doi: 10.1080/15592294.2022.2044127 (PMC9620995; doi:10.1080/15592294.2022.2044127)
Supplement: Supplemental Material [file KEPI_A_2044127_SM6710.zip › supplementary/Supplementary_figures.docx]

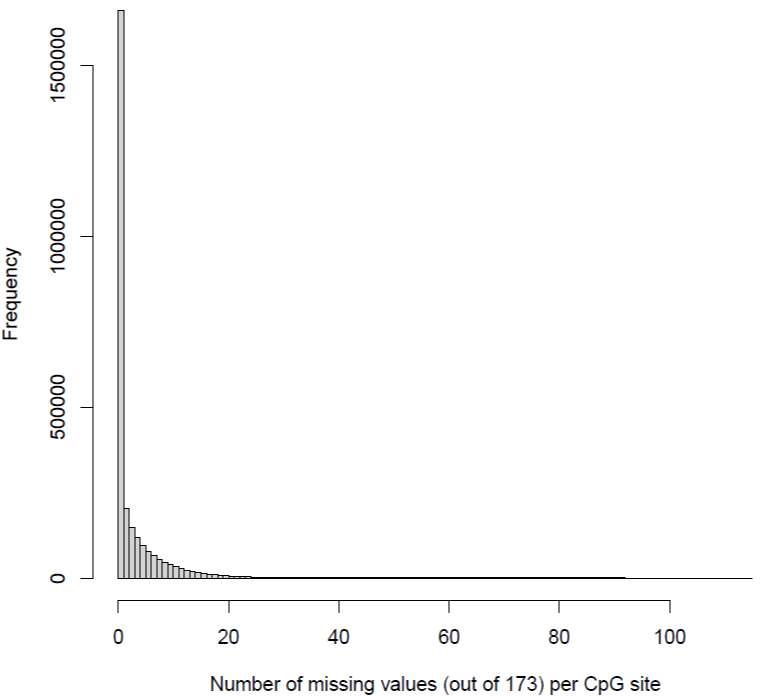


Supplementary Figure 1: A histogram of the number of missing (coverage = 0) values per CpG site out of a total number of 173 samples. A small fraction (2.6 %) of the CpG sites had more than 20 missing values. This histogram was created for the approx. 2.7 million CpG sites that remained after quality control and coverage filtering steps (i.e. those CpG sites that are part of the differential methylation analysis).


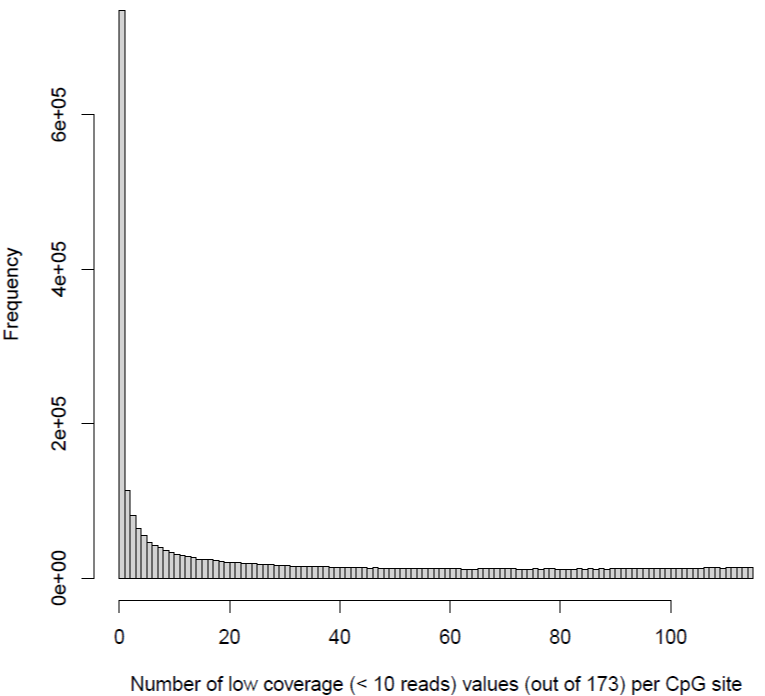


Supplementary Figure 2: A histogram of the number of low-coverage (coverage < 10 reads) values per CpG site out of a total number of 173 samples. This histogram was created for the approx. 2.7 million CpG sites that remained after quality control and coverage filtering steps (i.e. those CpG sites that are part of the differential methylation analysis).


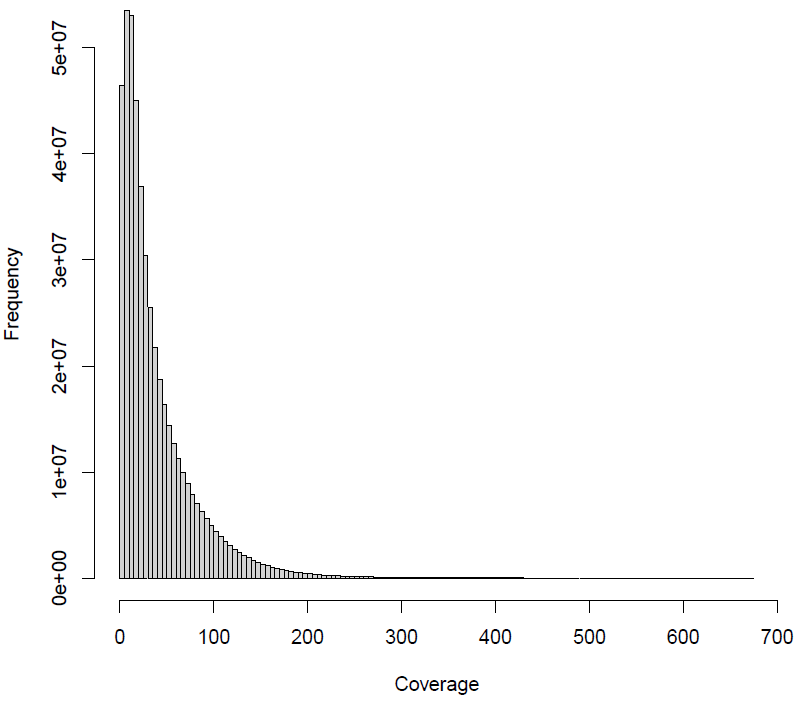


Supplementary Figure 3: The coverage distribution across the whole set of measurements that were part of the analysis (173 samples × approx. 2.7 million CpG sites that remained after quality control and coverage filtering steps)


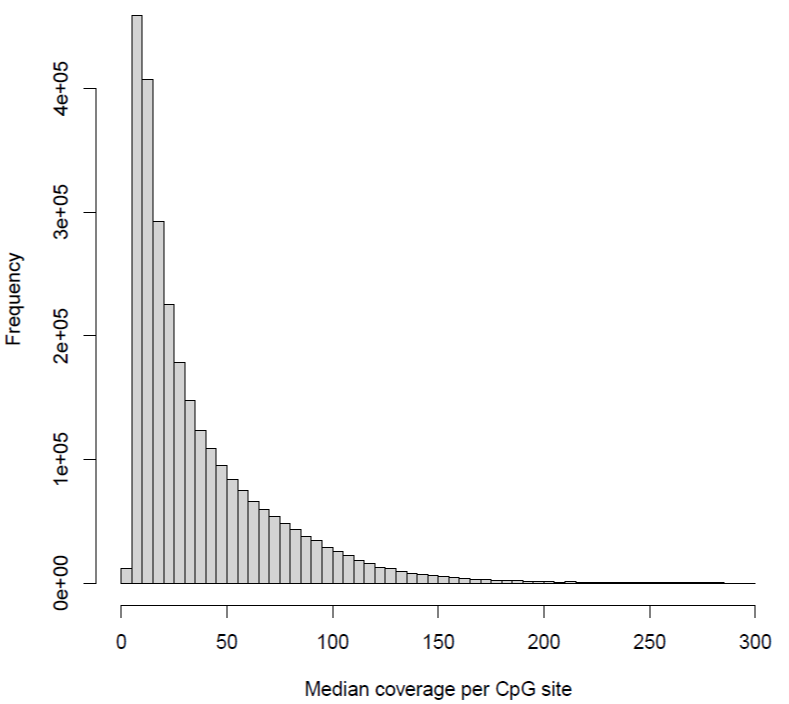


Supplementary Figure 4: A histogram of the median coverage of each CpG site. This histogram was created for the approx. 2.7 million CpG sites that remained after quality control and coverage filtering steps (i.e. those CpG sites that are part of the differential methylation analysis).
