# Supplementary material for: Permutation-based significance analysis reduces the type 1 error rate in bisulphite sequencing data analysis of human umbilical cord blood samples
Source: Epigenetics. 2022 Mar 4;17(12):1608–27. doi: 10.1080/15592294.2022.2044127 (PMC9620995; doi:10.1080/15592294.2022.2044127)
Supplement: Supplemental Material [file KEPI_A_2044127_SM6710.zip › supplementary/Supplementary_Table_2.docx]

Supplementary Table 2: Comparison between results from models that included 2,3, or 10 PCs. Here, correlations are Pearson correlations. DMCs are defined as CpG sites with Benjamini-Hochberg corrected P value < 0.05 (before spatial adjustment) and CpGs within candidate DMRs are all CpG sites with empirically FDR-controlled spatially adjusted P value < 0.05. A permutation analysis was only performed for one variable (sex) for the models with 3 and 10 principal components (therefore overlaps between candidate DMRs are not shown for other variables). The percentages are percentages of the original detections that are reported in the main text Table 1 (from the model that included 2 PCs).

|  | Correlation between log_10_(P values), 2PC vs. 3PC model | Correlation between log_10_(P values), 2PC vs. 10PC model | DMCs, overlap between 2PC and 3PC model | DMCs, overlap between 2PC and 10PC model |
| --- | --- | --- | --- | --- |
| Age, mother | 0.996 | 0.937 | - | - |
| Apgar points low | 0.994 | 0.912 | 2 (100 %) | 1 (50 %) |
| Birth weight | 0.981 | 0.849 | - | - |
| BMI, mother | 0.992 | 0.944 | - | - |
| C-section | 0.995 | 0.945 | - | - |
| Earlier miscarriage(s) | 0.995 | 0.971 | - | - |
| Epidural anaesthetic | 0.946 | 0.863 | - | - |
| Gestational weight gain, mother | 0.997 | 0.927 | - | - |
| Height, mother | 0.993 | 0.907 | 3 (100 %) | 2 (66.7 %) |
| Induced labor | 0.990 | 0.938 | - | - |
| Insulin-treated diabetes, mother | 0.973 | 0.901 | 4 (40 %) | 0 |
| Month of birth (cosine transformed) | 0.996 | 0.941 | - | - |
| Sex | 0.980 | 0.871 | 1315 (92.2 %) DMCs, 5872 (92.8 %) CpGs within candidate DMRs | 889 (62.3 %) DMCs, 5288 (83.5 %) CpGs within candidate DMRs |
| Smoking during pregnancy, mother | 0.994 | 0.961 | 1 (100 %) | 0 |
| Year | 0.996 | 0.926 | 6 (100 %) | 1 (16.7 %) |
